# An asthma self-management program based on WeChat to improve asthma control and quality of life: a randomized controlled trial

**DOI:** 10.3389/falgy.2025.1503597

**Published:** 2025-03-05

**Authors:** Shanshan Xu, Zhihui Song, Xiao Cheng, Jiawei Wang

**Affiliations:** Department of Pharmacy, Beijing Tongren Hospital, Capital Medical University, Beijing, China

**Keywords:** mobile phones, asthma, asthma control, WeChat application, self-management

## Abstract

**Background:**

Few studies have tested the feasibility and efficacy of WeChat-based asthma self-management, which supports patients in managing their asthma via mobile phone. We developed an intervention program based on the WeChat Mini program to support self-management. We evaluated the effectiveness and feasibility of improving asthma control and quality of life in patients with asthma.

**Methods:**

Fifty and 53 patients were randomized into the control and WeChat groups, respectively, to receive traditional interventions and interventions based on the WeChat Mini program. We conducted the intervention for three months and then observed for three months.

**Results:**

At the end of the third month, the Asthma Control Test (ACT) scores of the WeChat group were greater than those of the control group (*P* = 0.003), and the ACT scores of the two groups were significantly higher than those at baseline (*P* = 0.028; *P* < 0.001). At the end of the sixth month, the control group was not significantly different from the baseline group (*P* = 1.000), but the WeChat group was significantly different (*P* < 0.001). The ACT scores of the WeChat group were higher than those of the control group (*P* = 0.001). The ACT scores of the WeChat group were lower than those of the third month, but the difference was insignificant (*P* = 0.214). For asthma self-management and quality of life, the WeChat group improved more at the end of the third and sixth months (all *P* < 0.001).

**Conclusion:**

Implementing an asthma self-management program based on the WeChat application is effective in helping patients with asthma improve their asthma control and quality of life.

## 1 Introduction

Asthma is a serious global health problem that affects all age groups (1). The overall prevalence of asthma is 4.2%, and the prevalence of asthma with airflow limitation is 1.1% among Chinese adults aged >20 years (2). The goals of asthma management are to achieve good symptom control, relieve symptoms when they occur, and minimize the risk of exacerbations, asthma-related death, persistent airflow limitation, and treatment side effects (3). Currently, asthma control is low in China. Among people with asthma in China, 15.5% reported at least one emergency room visit, and 7.2% reported at least one hospital admission (2). Supported self-management of asthma can improve patient outcomes, such as asthma control, quality of life, medication adherence, and markers of asthma control, and several countries have developed guidelines for the self-management of patients with asthma (4–8). The interventions of medical staff, including pharmacists, play an important role in helping patients achieve effective self-management. A prospective intervention study also showed that self-management education provided by community pharmacists significantly improved patients’ knowledge, beliefs, and attitudes toward asthma medications and adherence to asthma control (9). The ADolescent Adherence Patient Tool (ADAPT) study also proved that a community pharmacy-based mHealth intervention could improve patients’ medication adherence and asthma control (10).

With the development of the Internet and mobile devices, many mobile health applications (apps) are available to facilitate self-management in patients with chronic diseases. Several studies have shown that apps can significantly improve clinically relevant indicators of chronic disease management or help specified users achieve specific goals, including effectiveness, efficiency, and satisfaction. A meta-analysis of five randomized trials indicated that tele-monitoring solutions including WeChat social media might be considered as sustainable strategies to implement Health-Related Quality of Life (HRQoL) in the long-term management of older patients with Chronic Obstructive Pulmonary Disease (COPD) (11). A two-arm, randomized, follow-up investigation reported that receiving daily WeChat services on one's cell phone could improve adherence to corticosteroid nasal spray treatment in chronic rhinosinusitis (CRS) patients after functional endoscopic sinus surgery (FESS) (12). However, many mobile health interventions have failed during clinical implementation. For example, neither the study by Klausen et al. nor by Michael et al. (13, 14) reported an improvement in HRQoL in pediatric patients with congenital heart disease (CHD) after a web-based intervention. More research is needed to confirm the benefits of mobile health applications and determine what makes a given mobile app intervention effective in improving chronic disease management.

WeChat is China's most popular social network, with more than 1 billion monthly active users (15). It is a free, cross-platform app widely used for sending text, voice, video, and photos, and users can conveniently subscribe to public or official accounts on the WeChat platform to acquire selected news or information. Because of the above advantages, WeChat has been widely used in managing chronic diseases, such as hypertension, diabetes, coronary heart disease, and cancer, as an effective technological method (16–18). The effect of WeChat intervention on the health behavior of patients with respiratory diseases has been confirmed by researchers (19, 20). Currently, limited and inconclusive evidence supports asthma self-management using the WeChat application. To overcome this dilemma, we developed the WeChat Mini program as an educational and follow-up tool to conduct a pharmacist-led intervention on the self-management of patients with asthma to evaluate whether it could improve the self-management ability of patients and the control of asthma.

## 2 Materials and methods

### 2.1 Study design and participants

This study was approved by the ethics review board of Beijing Tongren Hospital (TRECKY2020-106) on 3rd September, 2020, and was registered with the Chinese Clinical Trial Registry (ChiCTR2300075425). All study participants provided informed written consent, and the study followed the guidelines established by the Declaration of Helsinki. We have adhered to the CONSORT guidelines for writing this manuscript (21).

This was a 6-month prospective, two-arm, open-label, non-blinded, randomized controlled trial conducted by pharmacists at a large teaching hospital in China. The intervention period was three months because of the health habit formation and physiological adaptations to exercise during this period (22, 23); the remaining three months constituted the observation period. A random number generator (https://www.random.org/) was used to determine the study arm assignments of participants. The patients were then randomly divided into WeChat and control groups at a ratio of 1:1. This study adheres to the CONSORT guidelines, and a CONSORT checklist is provided in Additional File 1.

We planned to enroll 120 patients in this study, calculated based on a completely random design using the sample size formula to compare the mean of two independent samples. According to the nature of the trial design, neither pharmacists nor participants were blinded to the intervention.

The inclusion criteria were as follows: (1) ≥18 years of age, ability to complete the questionnaire by themselves, and willingness to accept self-management intervention; (2) confirmed diagnosis of moderate or severe asthma according to the guidelines of respiratory physicians (1); (3) received standard treatments according to their asthma grade during the previous 6 months; and (4) had a smartphone and used WeChat for effective communication. The exclusion criteria were patients who (1) had serious cardiovascular, hepatic, renal, or mental disorders, (2) were attending other self-management programs, and (3) were unable to communicate.

### 2.2 Development of the WeChat mini program “asthma self-management”

Before the intervention, respiratory clinicians, pharmacists, nurses, patients with asthma, and workers at a software company formed an expert team to discuss and develop the WeChat Mini program titled “Asthma Self-management”. The construction workflow of the WeChat Mini program is shown in Supplementary Figure S1. The core part of the program included asthma-related educational articles, videos of inhaler equipment, an asthma control test (ACT), an asthma diary card, and an online consultation. Asthma-related educational articles included asthma characteristics, treatment, smoking cessation, diet guidance, triggers, adherence improvement, differences between relievers and controllers, peak flow meter use, asthma therapy goals, and recognition and action when an asthma attack occurs. We uploaded all videos used for all common inhaler equipment, such as salmeterol fluticasone powder inhalers, budesonide, and formoterol powder inhalers. The Asthma Control Test (ACT) is a 5-item questionnaire for assessing asthma control. The asthma diary card includes records of asthma symptoms, peak expiratory flow (PEF) monitoring, and drug use. The patients could consult with pharmacists online at any time.

### 2.3 Interventions

All patients in the two groups received the same face-to-face education on asthma characteristics, treatment, use of inhaler equipment, filling in an asthma diary, use of a peak flow meter, importance of self-management, and other related education uploaded to the program by the pharmacist at the initial visit. The patients in the control group were given an asthma diary card handbook and instructed to refill it daily. They were also informed to revisit the pharmacy or receive a face-to-face interview of 20–30 min every month for the first 3 months. The pharmacist answered the questions and performed routine medication therapy for the patients when they revisited the pharmacy. All patients in the WeChat group were invited to subscribe to the WeChat Mini program “Asthma Self-management” on their smartphones and were introduced to how to use it by the pharmacist. The app featured four core functions to stimulate self-management and improve adherence. (1) To help monitor symptoms, patients were instructed to complete the asthma diary daily and the Asthma Control Test at least weekly. The pharmacist supervised the patient's situation by watching the completed content and receiving appropriate interventions or feedback, if necessary, by phone or via WeChat during the initial three-month phase. (2) There was an educational repository containing asthma-related educational knowledge, such as instructional videos about inhaler use, daily life management, and acute attack strategies for patients to view and learn. (3) Auto-reminder messages from the program were sent to patients to remind them to perform drug inhalation and complete an asthma diary every day. (4) There was a communication portal allowing patients to submit text/image/video queries to consult any questions anytime, and pharmacists were obligated to provide timely responses, suggestions and tailored educational materials. Additionally, all patients in the WeChat group were required to upload inhaler technique videos every three days post-baseline for pharmacist evaluation until competency was achieved. There were no interventions for the other group during the remaining 3 months of the observation period. All participants underwent baseline peak flow measurements at the initial visit.

### 2.4 Measures and outcomes

The study's primary outcome was asthma control, which was measured using the Asthma Control Test (ACT) scale. The ACT survey is a patient-completed questionnaire with five items assessing asthma symptoms (daytime and nocturnal), the use of rescue medications, and the effect of asthma on daily functioning. Each item includes five response options, corresponding to a 5-point Likert-type rating scale. When scoring the ACT survey, responses for each of the five items were summed to yield a score ranging from 5 to 25. In general, the higher the ACT score, in the range of 5–25, the better the control (24).

The secondary outcomes were pulmonary function (percentage of forced expiratory volume in 1 s predicted, FEV1%/predicted), Asthma Self-Management Behavior Questionnaire (ASMQ), and Asthma Quality of Life Questionnaire (AQLQ). The ASMQ is a patient-reported questionnaire to assess the self-management knowledge and skills of patients with asthma. We designed our study by referring to the ASMQ developed by Wei Chun (25) and the test of adherence to inhalers (TAI) (26). It comprises 28 items covering five dimensions (avoiding triggers, illness monitoring, identifying and managing acute symptoms, medication use and adherence, and seeing a doctor promptly). Each item was assessed using a 5-point Likert scale, with a total score ranging from 28 to 140 points, with higher scores indicating better self-management. The reliability and validity test results were 0.95, and Cronbach's *α* was 0.829. We used the Chinese Asthma Quality of Life Questionnaire (C-AQLQ) developed by Li F. et al. (27) based on the Juniper AQLQ to measure the problems that adults with asthma experience in their day-to-day lives; this questionnaire comprises 35 items covering 5 dimensions: activity limitation (12 items), symptoms (9 items), mental health (5 items), environmental stimuli (5 items), and health perceptions (4 items). The score ranged from 1 to 7 for each item, with a total possible score of 245 points, with a higher score indicating a better quality of life. The FEV1%/predicted ratio is an indicator of pulmonary function. A value ≥ 80% indicates normal pulmonary function. Other outcomes included emergency department (ED) visits and hospitalizations. The effectiveness of the WeChat Mini program was evaluated for all the above outcomes.

Demographic and sociological information about the patients in the two groups, including their age, sex, monthly family income, education level, contact information, smoking status, asthma medications, asthma severity (graded by medication level as GINA 2024), and asthma duration, was collected at the initial visit. The pharmacist collected the outcomes at baseline and 3 and 6 months. We compared the total scores of the primary and secondary outcomes and the number of ED visits or hospitalizations between the two groups.

### 2.5 Data collection and statistical analysis

An Excel spreadsheet was used to collect patient information. Values were assigned to each outcome, and the data were entered into a sheet. Two researchers entered and checked the data to ensure accuracy. Each participant will be assigned a unique study ID, and all personally identifiable information will be carefully de-identified before using the data.

Statistical analyses were performed using SPSS version 26 (Chicago, IL, USA). The results for each variable are shown as mean ± standard deviation for continuous variables and as frequency and percentage for categorical variables. Sex, education level, smoking status, asthma severity, monthly family income, and number of inhalations were analyzed using the chi-squared test. Age was analyzed using the independent-sample *t*-test, while the duration of asthma and the number of drugs used for asthma treatment were analyzed by nonparametric tests (Mann–Whitney *U*-test). To obtain insights into the correlation between asthma self-management behavior and quality of life and which ASMQ domains lead to improved quality of life, we used generalized linear mixed models with the improvement in the AQLQ score during follow-up as a dependent variable and the improvement in separate ASMQ domains as the independent variable. All reported *p*-values were two-sided, with alpha set at a significance level of 0.05.

## 3 Results

**Figure 1:**
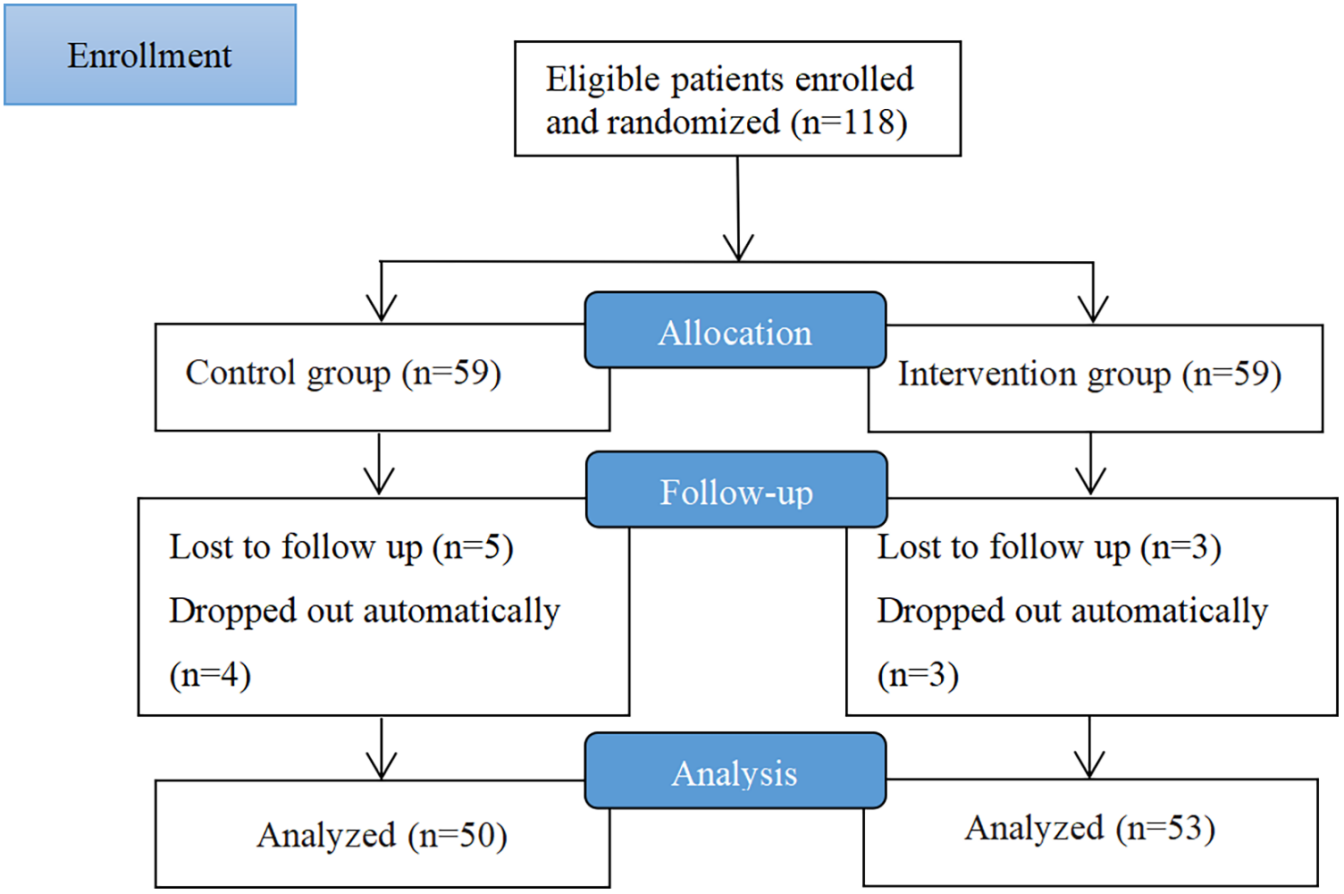
Flow chart of the study.

One hundred and eighteen participants were enrolled in the study, with 59 in each group. After 3 months of follow-up, 103 participants completed the study and were analyzed 50 patients in the control group and 53 patients in the WeChat group. Nine and seven participants in the control and WeChat groups, respectively, were lost to follow-up. Of 103 patients, 54 were female and 49 were male. There was no significant difference in the sex ratio between the two groups (*P* = 0.933). The mean age was 44.3 years in the control group and 42.89 years in the WeChat group, and the mean durations of asthma were 14.46 and 12.92 years in the control group and WeChat group, respectively. Most patients had severe asthma, accounting for 70% (35/50) and 66.04% (35/53) of patients in the control and WeChat groups, respectively. The proportion of current smokers in the control group (18%, 9/50) was comparable to that of the WeChat group (11.32%, 6/53). The monthly family income of most patients was between 1,500 and 4,000 dollars (control group, 48% [24/50]; WeChat group, 45.28% [24/53]). Figure 1 shows a flow chart describing the study process in detail. There were no statistically significant differences in the baseline characteristics between the two groups (Table 1). Baseline characteristics did not influence the outcomes.

**Table 1:** Basic demographic characteristics and measurements of the two groups.

Parameter	Control group (*n* = 50)	WeChat group (*n* = 53)	*P* value
Gender, male, *n* (%)	24 (48)	25 (47.17)	0.933
Age, years, mean (SD)	44.3 (10.25)	42.89 (10.82)	0.251
Duration of asthma, years, mean (SD)	14.46 (7.54)	12.92 (7.62)	0.237
Smoking status			0.572
Never, *n* (%)	28 (56)	30 (56.60)	
Ex, *n* (%)	13 (26)	17 (32.08)	
Current, *n* (%)	9 (18)	6 (11.32)	
Asthma severity			0.864
Moderate, Step-3 treatment, *n* (%)	15 (30)	18 (33.96)	
Severe, Step-4 treatment, *n* (%)	29 (58)	30 (56.60)	
Severe, Step-5 treatment, *n* (%)	6 (12)	5 (9.43)	
Education level			0.785
Middle school and below, *n* (%)	11 (22)	10 (18.87)	
High school, *n* (%)	16 (32)	15 (28.30)	
College degree and above, *n* (%)	23 (46)	28 (52.83)	
Monthly family income, mean (SD), $	3,192 (1,785.62)	3,336 (1,752.05)	1.000
Monthly family income, *n* (%)			0.827
≤ 1,500 $	8 (16)	7 (13.21)	
1,500∼4,000 $	24 (48)	24 (45.28)	
≥ 4,000 $	18 (36)	22 (41.51)	
Number of drugs used for asthma, mean (SD)	2.18 (0.77)	2.09 (0.69)	0.494
Measurements			
ACT, mean (SD)	17.78 (4.26)	17.98 (4.28)	0.756
ASMQ, mean (SD)	80.54 (11.85)	81.26 (14.96)	0.076
AQLQ, mean (SD)	178.52 (17.50)	165.08 (17.57)	0.876
FEV1% predicted, mean (SD)	60.98 (13.84)	63.21 (12.58)	0.349

### 3.1 Asthma control

**Figure 2:**
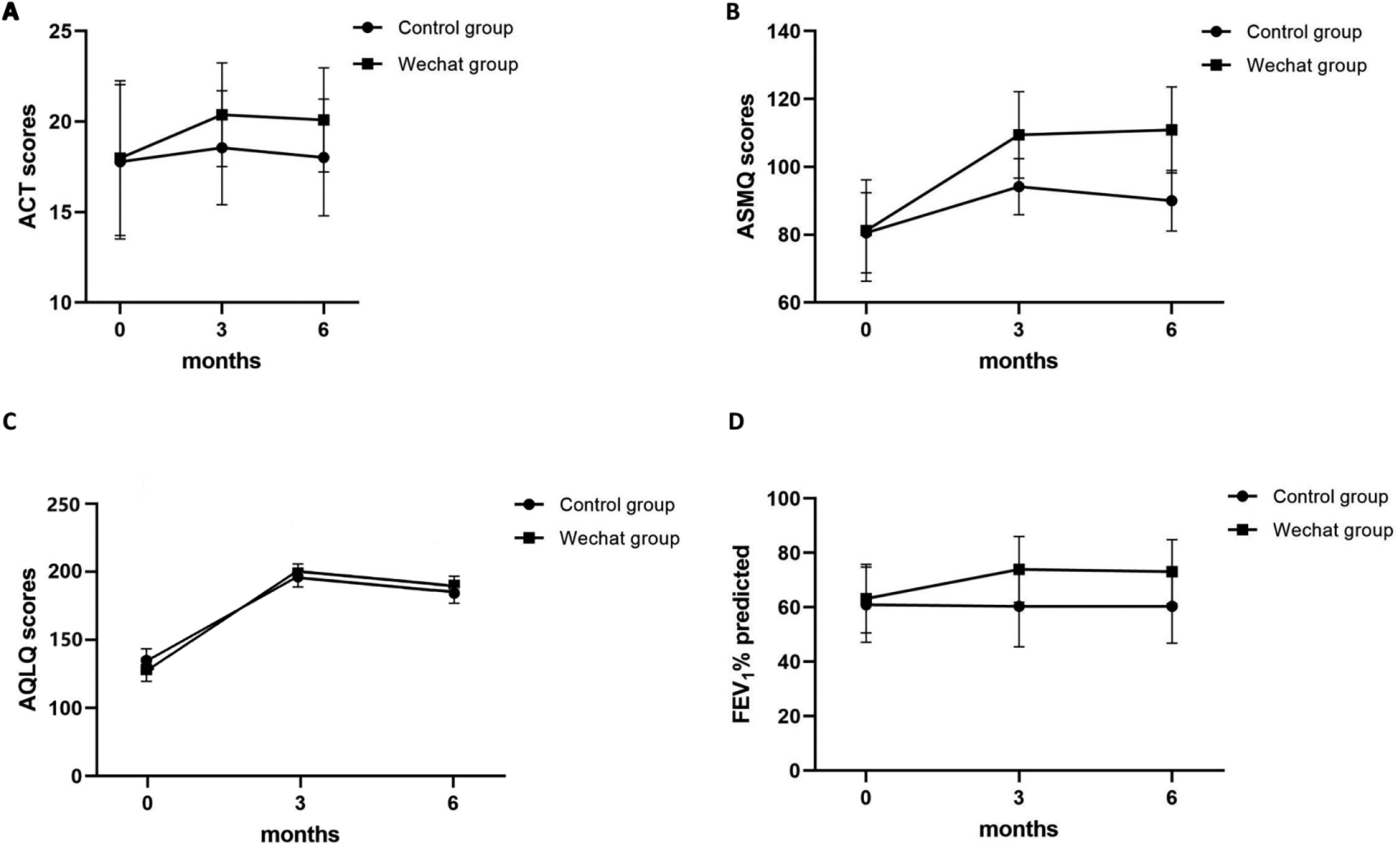
The changing trend of **(A)** ACT, **(B)** ASMQ score, **(C)** AQLQ score, and **(D)** FEV1%/predicted over time.

ACT was used to assess the level of asthma control. At baseline, 58% (29/50) and 58.49% (31/53) of each group had ACT scores less than 20; these patients were considered to have uncontrolled asthma. Consequently, 42% (21/50) and 41.51% (22/53) of the patients in each group had well-controlled asthma, with ACT scores of ≥20. The changing trend in ACT scores is shown in Figure 2A. After three months, the ACT scores in the control and WeChat groups increased from 17.78 and 17.98 to 18.56 and 20.38, respectively, and both groups presented increased scores compared to baseline (*P* = 0.028 and *P* < 0.001, respectively; Table 2). After six months of follow-up, the ACT score in the control group changed to 18.02, which was not different from the baseline score (*P* = 1.000). However, the ACT score in the WeChat group changed to 20.09, which was a significant difference (*P* < 0.001). The difference between 3 and 6 months was not statistically significant in the WeChat group (*P* = 0.214) but was statistically significant in the control group (*P* = 0.003). Between-group differences were observed at 3 and 6 months (*P* = 0.003 and *P* = 0.001, respectively; Table 3).

**Table 2:** Comparison of outcomes between the two groups before and after the intervention.

Group	Outcomes	3-month visit compared with baseline		6-month visit compared with baseline		6-month compared with 3-month visit	
		Mean difference (95% CI)	*P* value	Mean difference (95% CI)	*P* value	Mean difference (95% CI)	*P* value
Control group	ACT	−0.78(−1.50, −0.07)	0.028	−0.24 (−1.02, 0.54)	1.000	0.54 (0.15, 0.93)	0.003
	ASMQ	−13.62 (−16.31, −10.93)	<0.001	−9.52 (12.55, −6.49)	<0.001	4.10 (2.59, 5.61)	<0.001
	AQLQ	−15.16 (−19.84, −10.48)	<0.001	−10.88 (−15.38, −6.38)	<0.001	4.28 (2.36, 6.20)	<0.001
	FEV1% predicted	−2.14 (−8.26, 3.98)	1.000	−0.98 (−5.86, 3.90)	1.000	.16 (−2.80, 5.12)	1.000
WeChat group	ACT	−2.34 (−3.09, −1.70)	<0.001	−2.11 (−2.87, −1.36)	<0.001	0.28 (−0.10, 0.66)	0.214
	ASMQ	−28.64 (31.59, −25.70)	<0.001	−30.87 (−33.48, −28.26)	<0.001	−2.23 (0.76, 3.70)	0.001
	AQLQ	−29.81 (−34.36, −25.27)	<0.001	−27.15 (−31.52, −22.78)	<0.001	2.66 (0.79, 4.53)	0.002
	FEV1% predicted	−10.72 (−16.66, −4.77)	<0.001	−9.93 (−14.66, −5.19)	<0.001	0.79 (−3.05, 4.64)	1.000

**Table 3:** Mean changes in outcomes from baseline to the end of the study.

Outcomes	Baseline	After intervention		F(P)
		3-month visit	6-month visit	
ACT, mean (SD)				
Control group (*n* = 50)	17.78 (4.26)	18.56 (3.16)	18.02 (3.23)	8.36 (< 0.001)
WeChat group (*n* = 53)	17.98 (4.28)	20.38 (2.86)	20.09 (2.87)	35.47 (< 0.001)
F (*P* value)	0.06 (0.812)	9.38 (0.003)	11.90 (0.001)	
ASMQ, mean (SD)				
Control group (*n* = 50)	80.54 (11.85)	94.16 (8.30)	90.06 (8.96)	73.19 (< 0.001)
WeChat group (*n* = 53)	81.26 (14.96)	109.91 (10.41)	112.13 (10.42)	366.93 (< 0.001)
F (P)	0.07 (0.087)	106.95 (< 0.001)	92.98 (< 0.001)	
AQLQ, mean (SD)				
Control group (*n* = 50)	178.52 (17.50)	197.34 (22.73)	180.22 (15.03)	35.92 (< 0.001)
WeChat group (*n* = 53)	165.08 (17.57)	194.89 (14.01)	192.23 (13.70)	127.05 (< 0.001)
F (P)	0.006 (0.94)	24.16 (< 0.001)	31.97 (< 0.001)	
FEV1% predicted, mean (SD)				
Control group (*n* = 50)	60.98 (13.84)	60.36 (14.93)	60.4 (13.63)	0.39 (0.679)
WeChat group (*n* = 53)	63.21 (12.58)	73.92 (12.16)	73.13 (11.79)	13.16 (< 0.001)
F (P)	0.73 (0.39)	17.16 (< 0.001)	21.62 (< 0.001)	

### 3.2 Asthma self-management

The ASMQ was used to assess the self-management of patients with asthma. The changing trend of the score is shown in Figure 2B. After 3 and 6 months of follow-up, the ASMQ score in the control group increased from 80.54 to 94.16 and 90.06, respectively, and increased from 81.26 to 109.91 and 112.13, respectively, in the WeChat group. Both groups presented increased ASMQ scores compared to those at baseline (all *P* ≤ 0.001; Table 2), but the WeChat group showed greater improvement than the control group did (both *P* < 0.001; Table 3). The differences between 3 and 6 months were also statistically significant in both groups (*P* < 0.001 and *P* = 0.001, respectively; Table 2).

Comparisons of the scores on the 5 dimensions of the ASMQ between the two groups are shown in Supplementary Table 1. In the control group, the scores on all 5 dimensions improved from baseline to 3 months (*P* < 0.001, *P* < 0.001, *P* < 0.001, *P* < 0.001, and *P* = 0.004) or 6 months after surgery except for seeing a doctor in a timely manner (*P* < 0.001, *P* < 0.001, *P* = 0.011, *P* = 0.002, and *P* = 0.62, respectively). The differences between 3 months and 6 months were not statistically significant for avoiding triggers or seeing a doctor promptly or on time (*P* = 0.252 and *P* = 0.797, respectively), while they were statistically significant for the other 3 dimensions (*P* = 0.002, *P* < 0.001, and *P* = 0.001, respectively). In the WeChat group, the scores on all 5 dimensions improved from baseline to 3 or 6 months (all *P* < 0.001). The differences between 3 and 6 months were only statistically significant for avoiding triggers and monitoring illness (*P* = 0.005 and *P* = 0.001, respectively). Between-group differences were observed at 3 and 6 months in 4 dimensions, except for preventing triggers (*P* = 0.857 and *P* = 0.431, respectively).

### 3.3 Asthma quality of life

The AQLQ was used to assess the quality of life of patients with asthma. The changing trend of the score is shown in Figure 2C. After 3 and 6 months of follow-up, the AQLQ score in the control group increased from 178.52 to 197.34 and 180.22, respectively, and increased from 165.08 to 194.89 and 192.23 in the WeChat group. The AQLQ score increased in both groups compared to that at baseline (all *P* < 0.001; Table 2), but the WeChat group showed greater improvement than the control group did (both *P* < 0.001; Table 3). The differences between 3 and 6 months were also statistically significant in both groups (*P* < 0.001 and *P* = 0.002, respectively; Table 2).

A comparison of the scores on 5 dimensions of the AQLQ between the two groups is shown in Supplementary Table 2. In the control group, the scores on all 5 dimensions improved from baseline to 3 months (*P* = 0.001, *P* < 0.001, *P* < 0.001, *P* = 0.004, and *P* = 0.008, respectively) or 6 months (*P* = 0.033, *P* = 0.002, *P* = 0.004, *P* = 0.056, and *P* = 0.008, respectively). The differences between 3 months and 6 months were statistically significant for activity limitation and symptoms (*P* = 0.042 and *P* < 0.001, respectively), while they were not statistically significant for the other 3 dimensions (*P* = 0.184, *P* = 0.127, and *P* = 1.000, respectively). In the WeChat group, the scores on all 5 dimensions improved from baseline to 3 or 6 months (all *P* < 0.001). The differences between 3 and 6 months were statistically significant only for health perceptions (*P* = 0.014). Between-group differences were observed at 3 and 6 months in 4 dimensions, except for activity limitation (*P* = 0.351 and *P* = 0.309, respectively).

### 3.4 Pulmonary function

The FEV1%/predicted was used to assess the pulmonary function of patients with asthma. The changing trend of the percentage of FEV1/predicted is shown in Figure 2D. After 3 and 6 months of follow-up, the AQLQ score improved in the WeChat group compared to that at baseline (both *P* < 0.001), while the control group did not improve (both *P* = 1.000; Table 2). Between-group differences were observed at 3 months and 6 months (both *P* < 0.001; Table 3). The differences between 3 months and 6 months were not statistically significant in either group (both *P* = 1.000; Table 2).

### 3.5 ED visits (or hospitalization)

Four patients visited the emergency department (ED) at least once, 2 patients who needed hospitalization because of an asthma attack in the control group, and only 1 patient visited the ED in the WeChat group. However, the two groups had no significant differences in the incidence of ED visits or hospitalization (*P* = 0.042).

### 3.6 The impact of asthma self-management behavior on quality of life

The results of the generalized linear mixed models showed that asthma self-management behavior was positively related to improvement in quality of life (AQLQ score difference = 0.37, 95% CI = 0.25–0.50; *P* < 0.001). Moreover, we assessed the correlation between the improvement in asthma quality of life and changes in the other ASMQ domains. The results of the generalized linear mixed models are shown in Supplementary Table 3. Improvements in three domains, namely, avoiding triggers, monitoring of illness, and medication use and adherence, during follow-up were positively related to improvements in quality of life (*P* < 0.001; *P* = 0.012; *P* < 0.001, respectively). The other two domains, identification and management of acute symptoms and seeing a doctor in a timely manner, showed a positive relationship with quality of life (*P* = 0.274 and *P* = 0.176, respectively).

## 4 Discussion

In this study, we evaluated the effectiveness of a pharmacist-led intervention program based on the WeChat application in improving the self-management of patients with asthma. This study demonstrated the following. First, the asthma self-management program based on the WeChat application significantly improved the asthma control of patients. Compared with the control group, the WeChat group maintained better asthma control over time. This may be because the WeChat Mini program provided continuous education on asthma management, including correct inhaler usage and timely symptom monitoring. Other studies have also shown that continuous self-management support can effectively improve asthma control. For example, a meta-analysis of 32 randomized controlled trials demonstrated that self management programs could improve lung function and self-efficacy in patients with asthma (28). Second, the WeChat group had higher ASMQ and AQLQ scores, indicating better self-management and quality of life. The WeChat Mini program's functions, such as the asthma diary, educational articles, and online consultation, enabled patients to better understand their condition, master self-management skills, and actively participate in treatment. The real-time feedback and guidance from pharmacists also enhanced patients’ confidence and ability to manage their asthma, which was consistent with the findings of previous studies on mobile health-based self-management support (20, 29). Third, the improvement in the FEV1%/predicted in the WeChat group suggested that the WeChat-based intervention had a positive impact on pulmonary function. This could be attributed to better asthma control and more regular medication use under the guidance of the WeChat Mini program. However, compared with other studies on pulmonary function improvement in patients with asthma, the magnitude of the improvement in this study needs further exploration, and the long term impact on pulmonary function also requires more research.

In our study, the WeChat group had better asthma-related scores (ACT, ASMQ, and AQLQ) and greater improvement in the percentage of FEV1/predicted than the traditional intervention group. This may show that WeChat can play a positive role in asthma self-management. The reasons may be as follows: (1) Inhalation therapy plays an important role in controlling asthma. Incorrect use of inhalers may increase the risk of uncontrolled asthma, increase treatment costs, and cause unwanted adverse effects (30). According to a previous report, instructions must be repeated at least 3 times to achieve effective inhalation (31). In the traditional intervention, the pharmacists demonstrated the use of inhalers for every patient at the initial visit. In the WeChat Mini program, we added videos of inhaler equipment, and the patients could watch and learn how to use inhalers correctly repeatedly. Moreover, an auto-reminder message was sent to each patient every day, and the patient's medication adherence greatly improved. The results of the generalized linear mixed models also demonstrated that medication use and adherence were related to improvement in quality of life. (2) An auto-reminder message was sent to each patient daily to complete the asthma diary card. The pharmacists could provide real-time health guidance and adjust the asthma action plan according to the patient's asthma and physical condition. (3) Healthy lifestyles and asthma knowledge, such as identifying and managing acute symptoms, are also very important for asthma self-management. The WeChat Mini program can act as an effective tool for changing patients’ unhealthy living habits and enhancing patients’ understanding of asthma through using asthma-related educational articles. Moreover, pharmacists could post new information about asthma through the WeChat Mini program. Mental disorders are associated with poorly controlled asthma. Patients learn more about the disease and will be more confident when confronted with asthma. With positive psychological stimulation, patients obtained more benefits throughout the treatment.

In this study, the results of the second trimester were achieved without any interventions by pharmacists, which meant that the WeChat-based self-management program was easily feasible for implementation. However, optimal self-management, including an action plan and support from regular health professional reviews, was also necessary.

Compared with other interventions based on mobile-health, the WeChat-based intervention in this study showed certain advantages in improving asthma-related outcomes. However, some studies have reported that telehealth interventions have limited benefits for chronic disease management (32, 33). This may be due to differences in intervention methods, patient population, and study designs. Our study provides new evidence for the effectiveness of WeChat-based interventions in asthma management, and indicates that more research is needed to determine the key factors for the success of mobile-health interventions.

This study has several limitations. First, the study was limited by its single-center nature, and the sample size was small, which might cause bias in the results. Second, the follow-up period was only six months, and the relatively short study period limited the analysis of the durability of improved outcomes. Third, all outcomes of the present study were based on self-reported outcome measures except the percentage of FEV1/predicted. This may lead to reporting bias, as patients’ self-evaluations may be affected by various factors. Future studies should consider adding more objective measurement methods to improve the reliability of the results. The last limitation is the voluntary phone use of the WeChat Mini program. To prevent nonuse, pharmacists receive instruction to contact patients via the chat function when patients rarely use it. Almost 15% of patients dropped out because they were unwilling to continue using the program. This also indicates that strategies are needed to improve patient acceptance and compliance with mobile-health interventions.

## 5 Conclusions

In this study, a new WeChat Mini program was established to intervene in the self-management of patients with asthma, and this program has been proven effective at improving asthma control, self-management behavior, quality of life, and pulmonary function. It provided a new approach for asthma management, with potential advantages in improving healthcare service efficiency and patient participation. The WeChat Mini program could play a central role in health management in China because it could overcome many traditional obstacles, such as traveling and scheduling, improve the delivery and effectiveness of healthcare services, and be well-integrated into society. However, more support and efforts from various aspects are still needed to promote the benefits of WeChat to eventually be widely used in improving public health. Future research should focus on conducting multicenter, large sample size, and long-term follow-up studies to confirm the generalizability of the findings. At the same time, efforts should be made to optimize the functions of the WeChat Mini - program, such as improving user experience, enhancing the accuracy of health guidance, and exploring more effective ways to improve patient compliance.

## Data Availability

The raw data supporting the conclusions of this article will be made available by the authors, without undue reservation.
