# Supplementary material for: An asthma self-management program based on WeChat to improve asthma control and quality of life: a randomized controlled trial
Source: Front Allergy. 2025 Mar 5;6:1503597. doi: 10.3389/falgy.2025.1503597 (PMC11919652; doi:10.3389/falgy.2025.1503597)
Supplement: Supplementary file 1 [file Datasheet1.pdf]

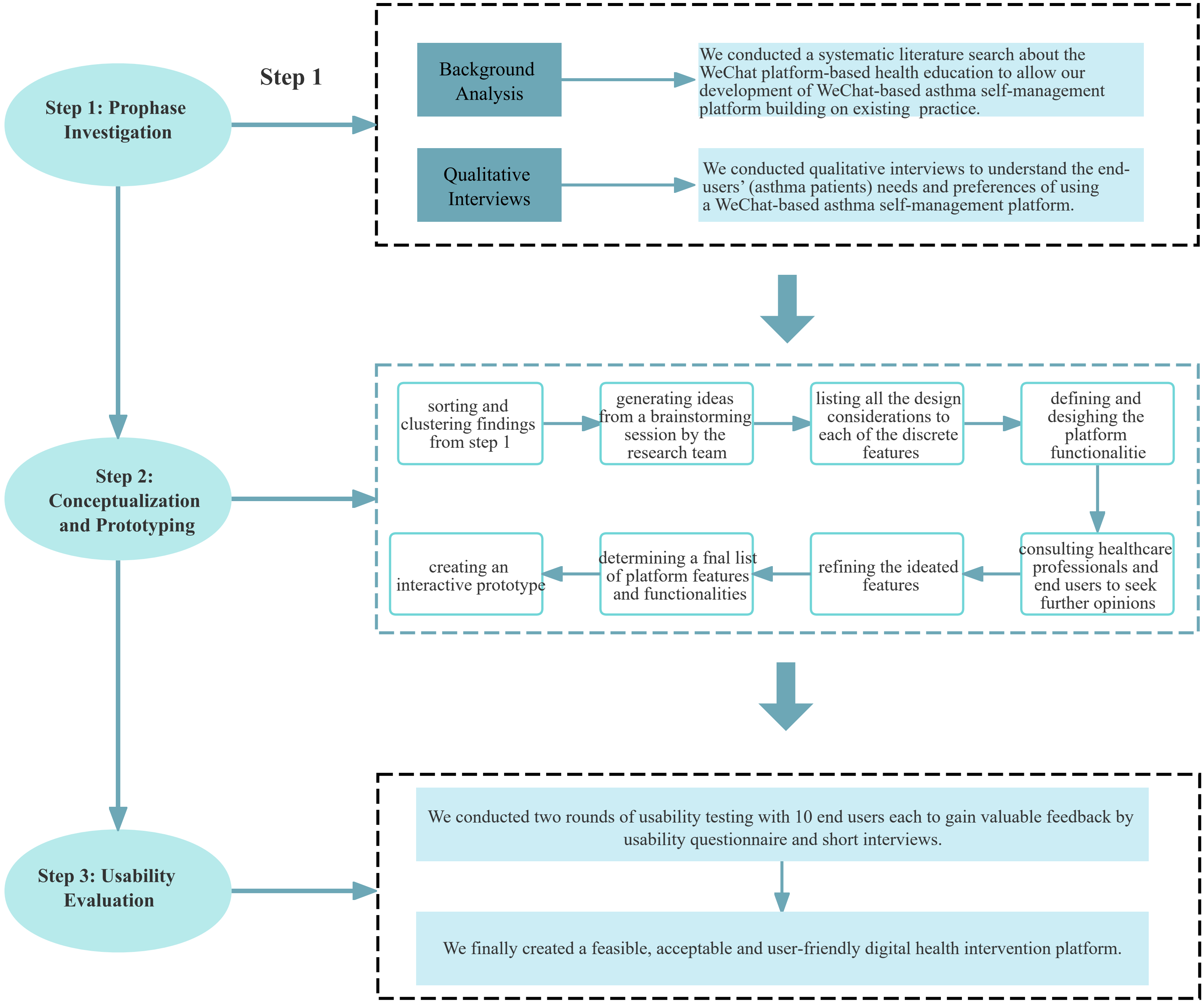

Fig S1. The construction workflow of WeChat Mini program.

Supplemental Table 1. Mean changes in 5 dimensions of ASMQ from the baseline to the end of the study.

		**Control group**					**WeChat group**				
		avoiding triggers	illness monitoring	identification and management of acute symptoms	medication use and adherence	seeing a doctor timely and on time	avoiding triggers	illness monitoring	identification and management of acute symptoms	medication use and adherence	seeing a doctor timely and on time
**Baseline, mean (SD)**		25.12 (0.90)	9.74 (0.31)	13.02 (0.40)	25.78 (0.96)	6.88 (0.24)	25.32 (0.87)	9.68 (0.30)	13.42 (0.39)	26.17 (0.93)	6.68 (0.23)
**3-month visit, mean (SD)**		28.86 (0.81)	11.94 (0.37)	14.86 (0.26)	31.06 (0.69)	7.44 (0.17)	28.66 (0.74)	13.85 (0.34)	17.93 (0.27)	40.43 (0.77)	9.04 (0.14)
**6-month visit, mean (SD)**		28.48 (0.76)	11.54 (0.35)	14.10 (0.28)	28.86 (0.80)	7.08 (0.15)	29.34 (0.79)	14.28 (0.36)	18.02 (0.26)	41.36 (0.67)	9.13 (0.16)
**within-group difference**	$P_{0,3}$[a]	< 0.001	< 0.001	< 0.001	< 0.001	0.004	< 0.001	< 0.001	< 0.001	< 0.001	< 0.001
	$P_{0,6}$[b]	< 0.001	< 0.001	0.011	0.002	0.62	< 0.001	< 0.001	< 0.001	< 0.001	< 0.001
	$P_{3,6}$[c]	0.252	0.002	< 0.001	0.001	0.797	0.005	0.001	1.000	0.286	0.797
**between-group differences**	F_1[d] (P)	0.03 (0.873)	0.02 (0.887)	0.49 (0.485)	0.09 (0.77)	0.37 (0.543)					
	F_2[e] (P)	0.33(0.857)	14.62 (<	68.60 (<	76.07 (< 0.001)	47.30 (<					

			0.001)	0.001)		0.001)
	F_3[f] (P)	0.63 (0.431)	28.87 (< 0.001)	102.99 (< 0.001)	154.10 (< 0.001)	101.55 (< 0.001)

[a]$P_{0,3}$: 3 months compared with baseline in each group.

[b]$P_{0,6}$: 6 months compared with baseline in each group.

[c]$P_{3,6}$: 6 months compared with 3 months in each group.

[d]F_1: Comparison between two groups at baseline.

[e]F_2: Comparison between two groups at the end of 3 months.

[f]F_3: Comparison between two groups at the end of 6 months.

Supplemental Table 2. Mean changes in 5 dimensions of AQLQ from the baseline to the end of the study.

		Control group					WeChat group				
		activity limitation	symptoms	mental health	environmental stimuli	health perceptions	activity limitation	symptoms	mental health	environmental stimuli	health perceptions
**Baseline, mean (SD)**		54.12 (1.67)	38.24 (1.07)	29.30 (0.76)	26.36 (0.67)	17.32 (0.55)	54.34 (1.62)	37.96 (1.04)	29.15 (0.74)	26.11 (0.65)	17.51 (0.53)
**3-month visit, mean (SD)**		58.14 (1.44)	43.12 (0.80)	32.38 (0.60)	28.24 (0.41)	18.62 (0.43)	60.02 (1.40)	47.19 (0.78)	36.28 (0.58)	30.28 (0.40)	21.11 (0.42)
**6-month visit, mean (SD)**		56.76 (1.56)	41.34 (0.73)	31.72 (0.59)	27.84 (0.43)	18.56 (0.42)	58.98 (1.51)	46.70 (0.71)	35.85 (0.58)	29.87 (0.42)	20.83 (0.41)
**within-group difference**	$P_{0,3}$[a]	0.001	< 0.001	< 0.001	0.004	0.008	< 0.001	< 0.001	< 0.001	< 0.001	< 0.001
	$P_{0,6}$[b]	0.033	0.002	0.004	0.056	0.008	< 0.001	< 0.001	< 0.001	< 0.001	< 0.001
	$P_{3,6}$[c]	0.042	< 0.001	0.184	0.127	1.000	0.166	0.619	0.609	0.091	0.014
**between-group differences**	F_1[d] (P)	0.01(0.925)	0.04 (0.853)	0.02 (0.888)	0.07 (0.791)	0.06 (0.805)					
	F_2 [e](P)	0.88 (0.351)	13.32 (< 0.001)	21.89 (< 0.001)	12.95 (< 0.001)	17.24 (< 0.001)					
	F_3[f] (P)	1.05 (0.309)	27.57 (<	24.99 (<	11.48 (0.001)	14.82 (<					

	0.001)	0.001)		0.001)

[a]$P_{0,3}$: 3 months compared with baseline in each group.

[b]$P_{0,6}$: 6 months compared with baseline in each group.

[c]$P_{3,6}$: 6 months compared with 3 months in each group.

[d]F_1: Comparison between two groups at baseline.

[e]F_2: Comparison between two groups at the end of 3 months.

[f]F_3: Comparison between two groups at the end of 6 months.

Supplemental Table 3. Relation between improvement in ASMQ domains and improvement in AQLQ

ASMQ domains	Mixed model effect sizes(β), mean (95% CI)	P value
avoiding triggers	-1.00 (-1.33, -0.66)	< 0.001
illness monitoring	0.86 (0.19, 1.52)	0.012
identification and management of acute symptoms	0.41 (-0.36, 1,27)	0.274
medication use and adherence	0.15 (.57, 1.17)	< 0.001
seeing a doctor timely and on time	.72 (-0.44, 2.40)	0.176

Appendix 1: General information questionnaire

This is a questionnaire about asthma. We will understand the direct or indirect relationship between your health status and asthma, to provide a scientific basis for effective control of asthma attacks and improve your health. Please answer all the questions on the questionnaire truthfully. We will keep all your personal information strictly confidential. Please put a " √ " in front of the appropriate answer. Thank you for your support and cooperation!

1. Sex: ☐ Male ☐ Female

2. Age: ___years

3. Ethnicity: ___

4. Place of residence: ___

5. First diagnosed with asthma in: ___

6. History of allergies: ___

7. Smoking history: ☐ Yes ☐ No

8. Marital status: ☐ Spouse ☐ No spouse

9. Literacy level: ☐ Elementary school and below ☐ Middle school ☐ Junior high School ☐ Junior college and high school ☐ University and above

10. Employment status: ☐ Unemployed ☐ Employed (☐ Employees of state-owned enterprises ☐ Employees of private enterprises ☐ Public institutions ☐ Farmers ☐ Individuals and others)

11. Monthly household income level: ☐ <1000 mouth ☐ 1000-3000 yuan ☐ 3000-5000 yuan ☐ 5000-10000 yuan ☐ >10000 yuan

12. Medical expenses are paid: ☐ Out-of-pocket expenses ☐ Public expenses ☐ Health insurance ☐ Commercial Insurance

Appendix 2: Asthma Control Test (ACT Rating Scale)

The following tests can help you with asthma assess how well your asthma is under control. Please answer as truthfully as possible.

Question 1

In the past 4 weeks, how often did your asthma keep you from getting as much done at work, school, or home?

A. All of the time

B. Most of the time

C. Some of the time

D. A little of the time

E. None of the time

Question 2

During the past 4 weeks, how often have you had shortness of breath?

A. More than once a day

B. Once a day

C. 3 to 6 times a week

D. Once or twice a week

E. Not at all

Question 3

During the past 4 weeks, how often did your asthma symptoms (wheezing, coughing, shortness of breath, chest tightness, or pain) wake you up at night or earlier than usual in the morning?

A. 4 or more nights a week

B. 2 to 3 nights a week

C. Once a week

D. Once or Twice

E. Not at all

Question 4

During the past 4 weeks, how often have you used your rescue inhaler or nebulizer medication (such as Salbutamol)?

A. 3 or more times per day

B. 1 or 2 times per day

C. 2 or 3 times per week

D. Once a week or less

E. Not at all

Question 5

How would you rate your asthma control during the past 4 weeks?

A. Not Controlled at all

B. Poorly Controlled

C. Somewhat Controlled

D. Well Controlled

E. Completely Controlled

Appendix 3: Asthma Self-Management Behavior Questionnaire (ASMQ)

There are 28 entries in this questionnaire, including five sections: Avoiding triggering (1-9), Illness monitoring Condition monitoring (10-13), Identifying and managing acute symptoms (14-17), Medication use and adherence (18-25), and Seeing a doctor promptly (26-28). Please select the appropriate option according to the actual situation in your daily life.

1) Avoiding triggers

1. I try not to be exposed to things that might trigger an asthma attack (e.g., cloth sofas, curtains, stuffed animals, pets, etc.).

2. Your family and friends are aware of your condition and assist in monitoring your self-management.

3. You try to avoid cold, spicy, and other stimulating foods, such as cold drinks, chili peppers, mustard, and so on.

4. You pay attention to the weather changes, increase or decrease clothing timely, and try to avoid letting yourself catch a cold.

5. When the outside air quality is poor, you will take protective measures (such as choosing suitable masks to go out).

6. You will stop or avoid people smoking around you.

7. You pay attention to regulating your emotions and avoid excessive excitement or tension.

8. You avoid strenuous exercise.

9. You spend at least 3 hours per week on aerobic exercise (e.g., swimming, jogging, etc.).

2) Illness monitoring

10. You pay attention to changes in your symptoms such as coughing, coughing fatigue, and

shortness of breath, and pay attention to changes in your condition by the observation points of the disease situation.

11. You use a peak flow meter to monitor your lung function on a regular daily basis. 12. You use the Asthma Control Test Questionnaire to determine how well your condition is controlled every month.

13. You keep an asthma diary.

3) Identifying and managing acute symptoms

14. You will perceive an asthma attack in time based on changes in symptoms such as shortness of breath, chest tightness, and coughing.

15. You carry your asthma medication with you whenever you go out, so you can use it right away when you have an asthma attack.

16. If you feel out of breath, you will rest quietly and calmly manage to relieve your symptoms.

17. Carry with you the phone number of someone you can contact immediately in case of an asthma attack.

4) Medication use and adherence

18. You will take your medication for a long time as required by your doctor.

19. You will take your medication as often as your doctor requires.

20. You will take the medication in the dosage prescribed by your doctor.

21. You will take your medication regularly at the times required by your doctor.

22. You know the names of the medications you take and what they do.

23. You are aware of the adverse effects of the drugs you take.

24. You will stop taking the medication on your own if your symptoms lessen or disappear.

25. You use the inhaler correctly

① Open the inhaler unit

② Slowly exhale the gas in your lungs before inhaling.

③ Place the suction nozzle in your mouth and wrap your lips around it tightly.

④ Inhale the medication deeply and smoothly with less than maximum inspiratory force.

⑤ Hold your breath for at least 5 to 10 seconds after inhaling.

⑥ Rinse your mouth promptly after inhaling.

5) Seeing a doctor promptly

26. If you don't have an asthma attack, you won't be able to keep up with your follow-up appointments.

27. You will have regular reviews as required by your doctor.

28. When asthma attacks are severe and cannot be improved by repeated inhalation of reliever medications, you will seek prompt medical attention at the hospital.

All questions contain the following five answers: A. All of the time, B. Most of the time, C. Some of the time, D. A little of the time, and E. None of the time

Appendix 4: Chinese Asthma Quality of Life Questionnaire (C-AQLQ)

The following are the most common daily activities. Please select five activities that you engage in most frequently in your daily life and write the number on the____ in the items 1 to 5.

(1) bicycling, (2) indoor cleaning, (3) pushing the bike, (4) jogging, (5) slow walking, (6) playing games with children, (7) singing, (8) dancing, (9) visiting relatives or friends, (10) taking the bus, (11) climbing stairs, (12) daily physical activity, (13) cleaning the floor, (14) chatting, (15) shopping.

Responses for items 1 to 7 are indicated in the following 7-point scale:

1-completely restricted, 2-extremely limited, 3-severely restricted, 4-moderately restricted, 5-mildly restricted, 6-seldom restrained, 7-not restricted

1. ____ In the past two weeks, to what degree has this activity been impacted by asthma: ()

2. ____ In the past two weeks, to what degree has this activity been impacted by asthma: ()

3. ____ In the past two weeks, to what degree has this activity been impacted by asthma: ()

4. ____ In the past two weeks, to what degree has this activity been impacted by asthma: ()

5. ____ In the past two weeks, to what degree has this activity been impacted by asthma: ()

6. In the past two weeks, to what degree have the activities that you must participate in been impacted by asthma: ()

7. In the past two weeks, to what degree have the activities that you should participate in been impacted by asthma: ()

Responses for items 8 to 35 are indicated on the following 7-point scale:

1-always, 2-very frequently, 3-frequently, 4-often, 5-sometimes, 6-occasionally, 7-never

8. In the past two weeks, you have walked away from cigarette smell in the surrounding environment:

()

9. In the past two weeks, you have walked away from perfume smell in the surrounding environment: ()

10. In the past two weeks, you have walked away from dust in the surrounding environment: ()

11. In the past two weeks, you have walked away from cooking fumes or soot smell in the surrounding environment: ()

12. In the past two weeks, you were forced to stay at home or go out because of the climate change or the smoke in the surrounding environment: ()

13. In the past two weeks, you have felt out of breath because of asthma: ()

14. In the past two weeks, the frequency of your asthma attack was: ()

15. In the past two weeks, you have felt uncomfortable because of a cough: ()

16. In the past two weeks, you have had a choking sensation or sense of impending doom: ()

17. In the past two weeks, you have felt chest tightness: ()

18. In the past two weeks, you have experienced difficulty in expiration: ()

19. In the past two weeks, you have had an asthma attack in the morning: ()

20. In the past two weeks, you have been awakened due to an asthma attack: ()

21. In the past two weeks, your sleep quality has been influenced by asthma attack: ()

22. In the past two weeks, you have felt sad or depressed because of asthma: ()

23. In the past two weeks, you lost confidence in treatment because of recurrent asthma attacks: ()

24. In the past two weeks, you have felt embarrassed by aerosol inhalation in front of other people: ()

25. In the past two weeks, you have worried that there were no drugs for asthma by your side: ()

26. In the past two weeks, you have worried about asthma attack: ()

27. In the past two weeks, you have had an asthma attack due to cigarette exposure: ()

28. In the past two weeks, you have had an asthma attack due to exposure to dust: ()

29. In the past two weeks, you have had an asthma attack due to exposure to cooking fumes or soot smell: ()

30. In the past two weeks, you have had an asthma attack due to exposure to perfume smells or strange odors: ()

31. In the past two weeks, you have had an asthma attack because of climate change or smoke in the surrounding environment: ()

32. In the past two weeks, you have worried about your current health condition because of asthma: ()

33. In the past two weeks, you have worried about your future health condition because of asthma: ()

34. In the past two weeks, you have worried about shortening of life due to asthma: ()

35. In the past two weeks, you have worried about your dependence on asthma sprays: ()
